# Tranexamic Acid: An Exceedingly Rare Cause of Anaphylaxis during Anaesthesia

**DOI:** 10.1155/2016/7828351

**Published:** 2016-10-31

**Authors:** R. A. Bansal, A. Nicholas, A. S. Bansal

**Affiliations:** Department of Immunology and Allergy, St Helier Hospital, Carshalton, Surrey SM5 1AA, UK

## Abstract

Tranexamic acid (TXA) allergy is extremely rare. An 80-year-old woman without prior exposure to TXA underwent elective knee replacement. Shortly after induction of anaesthesia and intravenous TXA, she developed hypotension, tachycardia, and facial erythema accompanied by a raised serum tryptase. Later, skin prick and intradermal testing confirmed positive responses to TXA in high dilution and with negative results to the other drugs used. While neuromuscular blocking agents, opiates, and antibiotics remain the most frequent cause of anaphylaxis during anaesthesia, allergy to TXA should always be borne in mind and requires skin testing for confirmation as there are presently no blood tests available.

## 1. Introduction

Anaphylaxis during anaesthesia is most frequently caused by allergy to the neuromuscular blocking agents followed by antibiotics and then opiates [1, 2]. Regardless of cause, an elevation and subsequent decline to normal levels of the serum mast cell tryptase are extremely helpful in confirming mast cell degranulation [3]. For many drugs used in anaesthesia, allergic reactivity requires skin testing as there are no useful in vitro tests available to confirm specific IgE binding. It is possible that the specific IgE binding of many anaesthetic agents often requires drug-protein interaction, much in the way of haptenation.

We describe an extremely rare case of anaphylaxis to tranexamic acid (TXA), reflected by clinical reactivity and raised mast cell tryptase and confirmed by skin testing. This is only the second time that this has been reported [4], but it should nevertheless be considered as a cause of cardiorespiratory distress during induction of anaesthesia (IOA). Informed consent was granted by the patient for her clinical details to be shared in this case report.

## 2. Case History

An 80-year-old woman of Indian origin underwent IOA with propofol, glycopyrronium, ketamine, ondansetron, midazolam, and fentanyl for an elective right total knee replacement. She also received cefuroxime and TXA as part of the normal protocol. She had never been exposed previously to tranexamic acid. The patient's past medical history included hypertension, type 2 diabetes, osteoarthritis of both knees and pancytopenia with bone marrow involvement requiring chemotherapy 6 years earlier. There was no history of atopy and she was currently taking Ramipril, Metformin, Alendronic Acid, and 3-monthly Vitamin B12 injections for pernicious anaemia.

Shortly after IOA, she developed a marked hypotension with tachycardia (blood pressure: 60/30 mmHg, heart rate: 170 beats per minute) that responded only slowly to the administration of IV noradrenaline and adrenaline. There was also a mild erythema of the face and chest noted. The serum tryptase levels confirmed mast cell degranulation with a serum level of 19.1 taken within 1 hour of the reaction occurring, which then declined to a normal level of 13.1 after 24 hours. She made a complete and full recovery and was discharged home well on day 7 after the operation. Allergic reactivity to cefuroxime, fentanyl, or propofol was considered causative of the reaction.

Two months after her reaction, skin prick testing revealed a moderately positive wheal of 5 mm with neat TXA, with entirely negative results for neat glycopyrronium, ketamine, propofol, and cefuroxime. Further negative tests occurred with 1/5 dilution of ondansetron and 1/10 dilution of fentanyl in normal saline. Subsequent intradermal testing showed negative results for 1/50 and 1/5 diluted glycopyrronium, 1/200 fentanyl, 1/5 propofol, and 1/5 cefuroxime. There were nonspecific reactions observed with ondansetron and ketamine, which produced a mild increase in wheal diameter at a 1/50 dilution that was negative at a 1/250 dilution.

However, intradermal testing with TXA produced a very significant 5-fold increase in wheal diameter with a 1/5 dilution, a 4-fold increase in diameter with 1/50 dilution, and a 2-fold increase with a 1/500 dilution. Skin and intradermal testing with neat TXA was negative in five healthy controls.

We attempted to detect TXA-specific IgE using ImmunoCAP allergen discs coated with neat TXA for one hour and then incubation with the patient's serum as per normal protocol on a Siemens ImmunoCAP 250. The results were negative (0.01 kUA/L) although the patient's total IgE was on the low side of the normal range at IgE 5.4 kUA/L.

The patient was advised to obtain a medic alert bracelet detailing her allergy to TXA and probable sensitivity to ondansetron and ketamine. She was also given the advice to have short period of premedication with antihistamine prior to a future attempt of knee replacement surgery.

## 3. Discussion

Our patient and her reaction have several similarities to the only other detailed report on TXA anaphylaxis [4]. In both cases anaphylactic reactivity occurred in elderly patients without previous atopy. Both patients developed oxygen desaturation, hypotension, tachycardia, and generalised erythema shortly after the TXA was infused. While serum tryptase levels were not raised at 30 and 120 minutes in the case reported by Lucas-Polomeni et al., blood tests showed a raised histamine level of 222 nmol/L (NR < 6 nmol/L). Skin testing 6 weeks after reaction in their case showed a positive 10 mm wheal with 20 mm erythema with neat TXA used in neat concentration. The patient later tolerated IOA with the same agents minus the TXA for a different procedure.

More recent reports on TXA reactivity have focused on retrospective cases. In the 5 cases reported by Imbesi et al. [5], many had systemic reactions following the parenteral or oral administration of TXA, but skin testing and blood tests to confirm allergic reactivity were not performed in 4 of the cases. In the fifth case skin prick and intradermal testing was performed with neat TXA. The application of Naranjo ADR probability scoring to these 5 cases suggests certain causality in 3 cases and probable causality in the other 2 cases. Our case had definite allergic reactivity with an elevated serum mast cell tryptase and intradermal testing positive at 1 in 500 dilutions.

While IgE-mediated type 1 hypersensitivity appears to be the most frequent manifestation of TXA allergy, T-cell mediated fixed drug eruption (FDE) has also been described. This was attributed to tranexamic acid and was unresponsive to graded introduction of TXA on 2 occasions, with rashes appearing with doses as low as 25 micrograms [6].

Although anaphylaxis during anaesthesia is frequently caused by NBA and antibiotics, we suggest that skin prick testing to neat TXA should be performed wherever this is used during IOA, to exclude this as a potential cause for allergic reactivity.
